# Time for a unified approach to medical ethics

**DOI:** 10.1186/1747-5341-4-13

**Published:** 2009-09-08

**Authors:** Shaheen E Lakhan, Elissa Hamlat, Turi McNamee, Cyndi Laird

**Affiliations:** 1 Global Neuroscience Initiative Foundation, Los Angeles, CA, USA

## Abstract

A code of ethics is used by individuals to justify their actions within an environment. Medical professionals require a keen understanding of specific ethical codes due to the potential consequences of their actions. Over the past thirty years there has been an increase in the scope and depth of ethics instruction in the medical profession; however the teaching of these codes is still highly variable. This inconsistency in implementation is problematic both for the medical practitioner and for the patient; without standardized training, neither party can be assured of the practitioner's overall depth of knowledge. Within the field of ethics certain principles have reached a consensus of importance. Incorporation of these concepts in meaningful ways via a consistent curriculum would provide students with an appropriate skill set for navigating their ethical environment. Moreover, this curriculum should also be extended to residents and professionals who may have missed formal ethical training. This would provide a consistent framework of knowledge for practitioners, creating a basis for clear judgment of complex issues.

## Introduction

Since the time of Hippocrates, new physicians have taken oaths to demonstrate their commitment to ethical principles essential to the medical field. More recently, it has been shown that approximately 98% of American medical graduates take such oaths at graduation [1]. One version of this modern Hippocratic Oath is taken by graduates of the Weill Medical College at Cornell University and can serve to summarize contemporary medical pledges [2]. Included in the pledge are vows to honor and sustain the profession of medicine, to pass on medical knowledge, to recognize limits and continue one's medical education, to seek help when needed, to never abandon patients, to live an honest life, to maintain a patient's privacy, to be a moral person, and to advocate for patients [3]. Similar ethical guidelines are also reflected in other medical documents, such as the Declarations of Geneva and Helsinki [4,5] and in legislation affecting those in the field, such as the Health Insurance Portability and Accountability Act (HIPAA) [6]. Many physicians promise to uphold these common ethical concerns. However, since the U.S. medical ethics curriculum is so varied, how can one gauge if medical students understand the oaths they take or the ethical and moral ramifications of the medical profession? Furthermore, are students ready to enter the world of medicine as ethical dilemmas continue to grow increasingly complex?

The past 30 years have seen a dramatic increase in ethical training given to US medical students but the varied nature of the curriculum makes evaluating these concerns difficult. Consensus is needed on the optimal implementations of ethics instruction, in order to unify teaching methods. Although this would require a great deal of concerted effort, a homogenization of medical ethics training would result in an increased trust in new doctors. Also, future improvements to medical programs will be far easier to accomplish.

### Medical ethics in medical school

In 1985, the Liaison Committee on Medical Education (LCME) sponsored by the American Association of Medical Colleges (AAMC) and the American Medical Association (AMA) added standards requiring that "ethical, behavioral, and socioeconomic subjects pertinent to medicine" be taught in medical programs across the US [7]. This increased interest in medical ethics had a variety of antecedents, among them "changes in the societal and ideological context of medical practice," "new technical capabilities (such as genetic screening or fetal organ donation)," and "a complex network of providers, insurers and health care monitors under new legal and regulatory control [8]." As a result, most medical schools added teaching of ethics to their curriculum, but without uniformity or consensus as to method or content.

For example, in 1985, 84% of responding schools taught "human values" in the first two years of medical school, while 34% taught these values during the last two years. By 1989, 43 of 127 American medical schools had required medical ethics courses, while 100 schools covered ethics within other courses [8]. This trend seems to have remained consistent over time. A comprehensive literature search by Eckles et al. in 2005 [9] demonstrated that although medical ethics instruction is almost universal, there remains no consensus on content, monitoring or quality of curricula. In fact, a large proportion of medical schools still integrate their medical ethics education into other courses.

In 2000, researchers DuBois and Burkemper [10] sought to find a common medical ethics curriculum. Specifically, they were looking to discover the strengths and weaknesses of any given school's program and to compare actual ethics courses with proposed "ideal" curricula. DuBois and Burkemper sent questionnaires to all American medical schools asking about their ethics instruction and requesting syllabi. They received responses from 72% of the schools, of which 79% of respondents said they required a formal ethics course. Of these, 84% provided their syllabi. Among responding schools there were a total of ten different course objectives, eight different teaching methods, thirty-nine different content areas, and six different methods of assessing students. The mean for a school was three objectives, four teaching methods, 13 content areas, and two methods of assessment. The specifics for each of these were listed, so that medical educators could then use these results to compare what they were teaching in their ethics classes. While this research did identify significant areas of overlap, overall discontinuities in course organization and application still offered cause for concern. Again, this concern rests in the current inability to accurately assess the universality and level of ethical competence within the medical community. With a unanimous curriculum, one may more confidently assume common exposure to the core values of medical ethics.

What then are these core values and what are the best ways to expose medical students to them? A start to answering the first question can be found in the *Standards for Accreditation of Medical Education Programs Leading to the M.D. Degree *in which the LCME outlines a section on medical ethics [7]. Section ED-23 states:

A medical school must teach medical ethics and human values, and require its students to exhibit scrupulous ethical principles in caring for patients, and in relating to patients' families and to others involved in patient care. Each school should assure that students receive instruction in appropriate medical ethics, human values, and communication skills before engaging in patient care activities. As students take on increasingly more active roles in patient care during their progression through the curriculum, adherence to ethical principles should be observed and evaluated, and reinforced through formal instructional efforts. In student-patient interactions there should be a means for identifying possible breaches of ethics in patient care, either through faculty/resident observation of the encounter, patient reporting, or some other appropriate method. 'Scrupulous ethical principles' imply characteristics like honesty, integrity, maintenance of confidentiality, and respect for patients, patients' families, other students, and other health professionals. The school's educational objectives may identify additional dimensions of ethical behavior to be exhibited in patient care settings.

Although more proscriptive than previous attempts to assure training of ethics in medical school curricula, it is still left to the medical school to determine which topics to address and how best to instruct their students. An ideal curricular approach can begin to form, however, by examining research from programs within the United States and abroad.

### Medical ethics in residency programs

Ethics training should not be limited to just medical students. While medical schools were incorporating medical ethics into their curricula, some residency programs were attempting to address the same issue. Residents may need an even deeper understanding of ethics, since they are often in difficult positions and must make critical decisions in patient care. Residents may absorb ethical lessons on a personal basis as these issues surface in daily practice.

Since 2007, the ACGME has included a set of core competencies in its Common Program Requirements for residents [11]. Among these competencies is professionalism, which includes the call for "an adherence to ethical principles." However, the common program requirements do not specify how ethical behavior should be taught, evaluated, or even remediated. These required competencies further reinforce the idea that unification of ethics education in all areas of medicine is needed.

In 1989, Perkins [12] discussed the greater need for ethics education among the resident population. Based on the work of the DeCamp Foundation published in the *New England Journal of Medicine *in 1985 [13], Perkins suggested that residents should be taught the moral aspects of medical practice; to know how to obtain informed, voluntary consent; to know what to do if a patient refuses recommended treatment; to know what to do about incompetent patients who are unable to understand information in a way that allows them to make and communicate a logical decision; to know when it is morally justified to withhold information; to know when breaching confidentiality is justified; to know how to manage patients with poor prognoses; and to know how to manage medical resources wisely.

### Defining medical ethics and guidelines for a curriculum

One of the first issues to be addressed in defining medical ethics is the lack of agreement on the primary goal of medical ethical education. In the previously discussed review, Eckles et al. [9] found two discrete goals for medical ethics training. The first was identified as "creating virtuous physicians." The second was identified as providing a skill set, which enabled physicians to navigate moral and ethical dilemmas. The concept of "creating virtuous physicians" is, in itself, somewhat problematic in that the quality of "virtue" is something that is typically instilled well before an individual reaches medical school and is therefore a duty perhaps best relegated to the medical schools' admissions committees. Creating a virtuous physician must begin with a virtuous individual. Admissions committees must seek to find those individuals that best exemplify "virtue" among their applicants if the school truly seeks to produce ethically-oriented physicians. The issue of which traits to seek out has been addressed in the works of Pellegrino [14] and include fidelity to trust, benevolence, intellectual honesty, courage, compassion, and truthfulness. Giordano and others [15] argue that the issue of ethics "rests upon the clinician as a moral agent" and advocate an "agent-based" virtue ethics, which emphasizes the integrity and emotional maturity of the clinician him- or herself.

Of the two goals, the "skill set" Eckles mentions is more of a curricular exercise. Reinforcement of the virtuous traits for which the students were selected should be encouraged, at a minimum. Several reasonable objectives have been outlined in the research and may serve as the basis of a unified curriculum. As Miles et al. [8] state, these are: "to enable physicians to examine and affirm their own personal and professional moral commitments, to teach physicians to recognize the humanistic and ethical aspects of medical careers, to equip physicians with a foundation of philosophical, social, and legal knowledge, to enable physicians to employ this knowledge in clinical reasoning, to equip physicians with the interactional skills needed to apply this insight, knowledge, and reasoning to human clinical care." These goals have been supported by other authors as well [8].

After determining the aims of ethics education, the question of implementation still remains. An obvious, but surprisingly limited, body of research addresses outcomes of ethical curricula already in place. Several conflicting conclusions have been found in regards to ethics education. Some studies posit that ethics education actually dampens physician sensitivity to ethical concerns, whereas others find a significant increase in moral reasoning capabilities [9]. The main source of this contradiction is methodological differences in measuring the ethical aptitude of physicians. This discrepancy undermines any real capability of inferring ideal teaching methods until analogous methods of evaluation are employed. In spite of this, some convergence does exist in specific methods of communicating ethics to medical students effectively.

One pedagogical tool which has repeatedly been found to be effective is the exploration of ethical concerns within the environment of small groups. In 1998, the *Journal of Medical Ethics *published a consensus statement from the teachers of medical ethics and law in UK medical schools in which they outline specific topics that ought to be addressed during medical training [16]. While not recommending specific instructional approaches, the importance of the clinical experience was emphasized in their recommended academic approach. The authors suggested a mix of small and large group discussion and recommended that instruction be case-based and at least partially student driven. The authors also proposed that there be at least one full time ethics instructor. They urged that the ethics curriculum begin early in medical school and be supported repeatedly; ideally it will be entirely integrated with the rest of the curriculum. Assessment of ethics curriculum should have the same importance as in any other area. The consensus statement mentioned seminars or classes in ethics specifically for the faculty, with formal assessment included.

In 2001, the Association of Teachers of Ethics and Law in Australia and New Zealand Medical Schools published a position statement outlining a core curriculum for the teaching of ethics [17]. Like the UK statement, it recommended a specific set of topics that should be taught to medical students, as well as a set of attitudes and skills. Emphasis was placed on the incorporation of experience and dialogue into the instructional approach, and the recommendation was made that at least some of the learning occur in small groups. This small group approach is also supported in Canada [18] and the United States [19]. The Australasian statement provides examples of integrating ethics instruction into medical education. These include ethics grand rounds, ethics journal clubs, "debriefing sessions" after the day to day practice of medicine, and scripted role-plays. It also lists strategies for assessment and evaluation of students. Some of these methods are written case reports, structured clinical exams, group assessment, and self or peer ratings. Assessment lets students know that ethics are valued by the medical school. The Australasian statement also addresses the limitations and challenges of incorporating ethics education; lack of qualified faculty to teach ethics and lack of time due to an already demanding curriculum.

There also seems to be consensus on the need to integrate the ethics curriculum into all four years of medical training [9]. A lack of continuity in ethics training may cause a medical student to lose focus on its importance, due largely to socialization and empathy erosion [20]. It has often been said that there is a "hidden curriculum" in medical school. Faculty may act unethically when practicing medicine and so act as negative role models for students. In one survey, almost half the students said they had been pressured to act unethically and 62% of medical students near the end of medical school "felt that their ethical principles had been seriously eroded or had disappeared" [21]. A longitudinal approach within medical education seems beneficial, with coursework and projects relevant to real-world medicine.

The University of Pittsburgh School of Medicine is one program which successfully utilizes this approach; students are offered opportunities for in-depth learning experiences throughout the four years of the medical curriculum. These opportunities include three initiatives: the Area of Concentration program, the Integrated Life Sciences courses, and the Scholarly Project. The first in this series is an elective, whereas the remaining two are fundamental parts of the required curriculum [19]. The Area of Concentration program and Scholarly Project are both student-driven. They each allow a student to choose and pursue a specific area of interest and research project, respectively, over the entirety of their four years at the institution. This allows students to confront ethical issues involved in research and medicine head on, in a way which is meaningful to them. Such techniques assimilate the learning of ethical and moral values more fully than a lecture or survey based class might [9,22], and in fact the benefits of this "deep approach" to learning have been previously documented [19].

The core values by consensus should be discussed with peer groups, keeping lecture or survey courses minimized in lieu of more meaningful presentation of real-world issues to students. A separate course devoted entirely to ethics would be the ideal. Applying these strategies, medical students will understand these core values better and come away with a fully developed skill set enabling them to deal proficiently with any concerns they may face in practice.

These strategies would enhance the evaluation of ethical skills as well. Using the defined elements as guidelines in a unified approach to ethics training would promote exposure of the *same *core values to all prospective doctors. With this unified method, measuring the efficacy of ethics education will become simpler. This is an important step for securing peace of mind for medical practitioners and for those individuals who depend upon them.

### Other options for learning: voluntary and compulsory

Doctors in practice now, depending on when they trained, may have missed out on formal training in ethics. However, they may still take continuing medical education (CME) courses in many related subjects. Some states are now adding specific CME requirements for re-licensure that include ethical components [23]. The State of California requires that most physicians and surgeons complete mandatory continuing education in the subjects of pain management and the treatment of terminally ill and dying patients [24]. In Florida, CME must include courses about domestic violence, end-of-life palliative care, and medical error [25]. In all states, medical boards can and do revoke medical licenses due to serious ethical lapses.

The AMA has a specific list of medical ethical principles, adopted in 2001, which they expect all physicians to uphold [26]. So, too, does the American College of Physicians (ACP) [27]. But to date, a specific uniform curriculum addressing these principles has yet to be implemented among medical schools, residency programs, or CME programs. Changing political, economic, and technological environments keep the ethical playing field in a constant state of flux. In order to ensure all practicing physicians are up to date, unified and centralized goals for continuing ethics training need to be implemented. By experiencing a universal system of ethics instruction from the start, modern physicians may have greater impetus to seek out continuing courses in ethics.

## Conclusion

A background in ethics has long been recognized as an important credential for medical professionals. Over the past thirty years, training in ethics has been incorporated into the medical curriculum, albeit with little regulation. Since recent advancements in medical care are associated with complex ethical issues, it is important that medical students, residents, and practicing physicians learn and understand ethics within a framework that is well-founded, rigorous, and longitudinally based.

Physicians must understand that ethics education will be a lifelong process throughout their medical career. While it may be safe to assume that most people who enter the profession will have their own moral code, physicians still need to keep up with the increasingly complex ethical dilemmas of modern medicine. A unified framework of ethics education ensures a measurable and accountable basis for the complex and far-reaching ethical issues present in the medical field.

Medical educators in Canada, the UK and Australia have reached consensus on aspects of ethics education in their respective countries including core values, curriculum, and teaching methods. The United States needs to reach consensus and then take the next step of national implementation. In 2006 the AMA launched ISTEP (Innovative Strategies for Transforming the Education of Physicians) [28]. This medical education research collaborative, which consists of 16 teams from 27 medical schools, is an example of the kind of collective needed to spearhead this process.

As a first step, medical educators in the United States may want to evaluate where the instillation of ethical values seems to have failed. Perhaps a review of reasons for revocation of medical licenses or expulsion from medical schools and residency programs would reveal areas in most urgent need of attention. Additionally, reviewing complaints brought against physicians by patients, colleagues, and others may demonstrate shortcomings in ethical instruction. A review by a committee of medical educators of subject material currently being taught at the medical student and resident level could produce a consensus statement as to which specific topics in ethics could be taught. Finally, comparing these topics to those already being addressed overseas could produce a core ethics curriculum which could be implemented into medical student and residency training, with a focus on small group and clinical case-based discussions. This could then be followed by rigorous evaluation of teaching methods and outcomes, with a goal of continuous process improvement. By doing so, we may be assured that certain specific fundamental ethical principles have been addressed during medical training, and know that failures in ethical behavior among practicing physicians are not due to shortcomings in medical education.

## Abbreviations

AAMC: Association of American Medical Colleges; ACGME: Accreditation Council for Graduate Medical Education; ACP: American College of Physicians; AMA: American Medical Association; CME: continuing medical education; HIPAA: Health Insurance Portability and Accountability Act; ISTEP: Innovative Strategies for Transforming the Education of Physicians; LCME: Liaison Committee on Medical Education.

## Competing interests

The authors declare that they have no competing interests.

## Authors' contributions

All authors participated in the preparation of the manuscript, and read and approved the final manuscript.

## About the authors

Dr. Lakhan is a medical scientist and executive director of the Global Neuroscience Initiative Foundation (GNIF). Ms. Hamlat and Ms. Laird serve as research assistants for the GNIF. Dr. McNamee is a research consultant at the GNIF, and an associate professor and internal medicine residency program director at Sanford School of Medicine of the University of South Dakota.
